# Cardiovascular magnetic resonance evaluation of aortic stenosis severity using single plane measurement of effective orifice area

**DOI:** 10.1186/1532-429X-14-23

**Published:** 2012-04-06

**Authors:** Julio Garcia, Oscar R Marrufo, Alfredo O Rodriguez, Eric Larose, Philippe Pibarot, Lyes Kadem

**Affiliations:** 1 Québec Heart & Lung Institute, Laval University, Québec, Canada; 2 Laboratory of Cardiovascular Fluid Dynamics, Concordia University, Montréal, Canada; 3 Department of Electrical Engineering, Universidad Autonoma Metropolitana, Mexico, DF, Mexico

## Abstract

**Background:**

Transthoracic echocardiography (TTE) is the standard method for the evaluation of the severity of aortic stenosis (AS). Valve effective orifice area (EOA) measured by the continuity equation is one of the most frequently used stenotic indices. However, TTE measurement of aortic valve EOA is not feasible or not reliable in a significant proportion of patients. Cardiovascular magnetic resonance (CMR) has emerged as a non-invasive alternative to evaluate EOA using velocity measurements. The objectives of this study were: 1) to validate a new CMR method using jet shear layer detection (JSLD) based on acoustical source term (AST) concept to estimate the valve EOA; 2) to introduce a simplified JSLD method not requiring vorticity field derivation.

**Methods and results:**

We performed an in vitro study where EOA was measured by CMR in 4 fixed stenoses (EOA = 0.48, 1.00, 1.38 and 2.11 cm^2^) under the same steady flow conditions (4-20 L/min). The in vivo study included eight (8) healthy subjects and 37 patients with mild to severe AS (0.72 cm^2 ^≤ EOA ≤ 1.71 cm^2^). All subjects underwent TTE and CMR examinations. EOA was determinated by TTE with the use of continuity equation method (TTE_CONT_). For CMR estimation of EOA, we used 3 methods: 1) Continuity equation (CMR_CONT_); 2) Shear layer detection (CMR_JSLD_), which was computed from the velocity field of a single CMR velocity profile at the peak systolic phase; 3) Single plane velocity truncation (CMR_SPVT_), which is a simplified version of CMR_JSLD _method. There was a good agreement between the EOAs obtained in vitro by the different CMR methods and the EOA predicted from the potential flow theory. In the in vivo study, there was good correlation and concordance between the EOA measured by the TTE_CONT _method versus those measured by each of the CMR methods: CMR_CONT _(r = 0.88), CMR_JSLD _(r = 0.93) and CMR_SPVT _(r = 0.93). The intra- and inter- observer variability of EOA measurements was 5 ± 5% and 9 ± 5% for TTE_CONT_, 2 ± 1% and 7 ± 5% for CMR_CONT_, 7 ± 5% and 8 ± 7% for CMR_JSLD_, 1 ± 2% and 3 ± 2% for CMR_SPVT_. When repeating image acquisition, reproducibility of measurements was 10 ± 8% and 12 ± 5% for TTE_CONT_, 9 ± 9% and 8 ± 8% for CMR_CONT_, 6 ± 5% and 7 ± 4% for CMR_JSLD _and 3 ± 2% and 2 ± 2% for CMR_SPVT_.

**Conclusion:**

There was an excellent agreement between the EOA estimated by the CMR_JSLD _or CMR_SPVT _methods and: 1) the theoretical EOA in vitro, and 2) the TTE_CONT _EOA in vivo. The CMR_SPVT _method was superior to the TTE and other CMR methods in terms of measurement variability. The novel CMR-based methods proposed in this study may be helpful to corroborate stenosis severity in patients for whom Doppler-echocardiography exam is inconclusive.

## Background

Transthoracic echocardiography (TTE) is the standard method for the evaluation of the severity of aortic stenosis (AS) [1]. One of the parameters that is most frequently used to assess AS severity is the aortic valve effective orifice area (EOA) determined by the continuity equation method. However, TTE measurements of EOA may not be feasible or reliable in a significant proportion of patients due to patients' characteristics, technical limitations or users' experience [1-4]. When the Doppler-echocardiographic measurements are not feasible or are discordant, it is important to confirm the stenosis severity with other, ideally non-invasive, diagnostic modalities.

Cardiovascular magnetic resonance (CMR) is a non-invasive, non-ionizing technique, with excellent temporal and spatial resolutions and superior measurement reproducibility. CMR may be used to measure the geometric (i.e. anatomic) orifice area (GOA) of the stenotic valve by planimetry [5-7]. However, the GOA is inferior to EOA to predict hemodynamic and clinical outcomes and its estimation may be difficult in heavily calcified valves [8,9]. CMR may be used to measure the EOA via the continuity equation. Several studies have shown that EOA obtained using CMR correlates well with the EOA obtained by TTE [10-13]. However, in a recent study performed by our group [13], we found that the resulting concordance between TTE and CMR for the EOA computed using the continuity equation is, in large part, due to the fact that the underestimation of A_LVOT _by TTE is compensated by an overestimation of VTI_LVOT_. We also discussed the potential variability in EOA values obtained using the continuity equation both by TTE and CMR as a result of the multitude of parameters to be measured. There is thus an important need for the development and validation of new simpler, more reproducible but still highly accurate CMR methods to estimate the EOA in AS patients. In a previous in vitro study, using particle image velocimetry measurements, we have shown that EOA can be directly determined using velocity measurements downstream of the stenosis and the application of acoustical source term concept (AST) [14]. Briefly, the fundamental idea behind this concept is that the flow jet created by the stenotic valve generates acoustic noise and the major sources of this sound generation can be determined by computing the acoustical source term. The acoustical source term is a function of the local velocity and the vorticity (a measure of the rate of rotation of fluid elements). Applied to AS, this means that the shear layer surrounding the orifice jet is a major source of acoustic noise. As a consequence, the limits of the jet-like zone downstream of the orifice, and therefore the EOA, can be determined using the AST maps without requiring the knowledge of the flow rate magnitude. In our previous in vitro study, we used particle image velocimetry, an optical technique that cannot be applied to the human body. Interestingly, it has been demonstrated that particle image velocimetry and phase-contrast velocity measure the same velocity map [15-19]. We can then hypothesize that the EOA of an AS could be determined using AST maps computed from CMR velocity measurements.

The objectives of this study are: 1) to extend the previous method for the determination of the EOA based on acoustical source term to velocity measurements obtained by CMR (here called Jet Shear Layer Detection method (JSLD)); 2) to introduce a simplified JSLD method not requiring vorticity field derivation. Both of the previously mentioned approaches require only a single velocity measurement (downstream of the AS) to determine the EOA. These new methods were evaluated both in vitro and in vivo. In the in vitro study, the EOAs determined by these new CMR methods were compared to the theoretical EOA predicted using the potential flow theory, whereas, in the in vivo study, they were compared to those obtained by standard TTE and CMR methods based on continuity equation.

## Methods

### In vitro study

The in vitro setup consisted of controllable pump generating steady flow (4 to 20 L/min), a compatible module with CMR magnet and a fluid reservoir. Four fixed circular stenoses (sharp-edge orifices with EOA = 0.48, 1.00, 1.38 and 2.11 cm^2^, with small aspect-ratios) were tested under the same steady flow conditions. Testing sharp-edge orifices, as models of fixed aortic stenosis, is a realistic approach since two (calcified thickened valve and thin fused valve) among the four more common morphological shapes of aortic stenoses can be represented by sharp-edge orifices [20]. Flow rate was measured with a Transonic flow probe 16A415 (accuracy: ± 4%, on full scale) connected to a T206 Transonic flow meter (Transonic, Ithaca, NY, USA) and was calibrated using a standard flow measuring method. A 65% saline and 35% glycerine (in volume) solution at room temperature was used to mimic viscous proprieties of blood at 37°C [21]. The use of such Newtonian fluid is justified in the context of aortic valve and ascending aorta [22-24]. A similar approach was used by others [25-27].

Each orifice was placed at the center of a clinical 3 Tesla magnetic resonance scanner with a dedicated phase-array receiver coil (Achieva, Philips Medical Systems, Best, the Netherlands). An ECG patient simulator (model 214B, DNI Nevada Inc, USA) was used to synchronize scanner gating. A standard examination was performed by initial acquisition of images in long-axis and short-axis planes for planning. Phase-contrast retrospective examination was performed in short-axis planes 12 mm upstream and 10 mm downstream of to the orifice plane. Imaging parameters consisted of: TR/TE of 17.99/3.97 ms, flip angle 15°, 50 phases, pixel spacing 1.25 mm, slice thickness 10 mm, acquisition matrix of 256 × 256 and encoding velocity (2 × maximal velocity).

A custom-made research application was developed using Matlab software (Mathworks, Natick, Ma, USA) to process and analyze in vitro and in vivo images [28].

### In vivo study

#### Patient population

Eight (8) healthy subjects and 37 patients with mild to severe AS (0.72 cm^2 ^≤ EOA ≤ 1.71 cm^2^) were included in this study. Exclusion criteria were: age < 21 years, LV ejection fraction < 50%, atrial fibrillation, mild mitral or aortic regurgitation, poor TTE imaging quality and standard contra-indications to magnetic resonance imaging. All patients provided written informed consent under the supervision of the institutional review board. AS severity classification followed American College of Cardiology/American Heart Association (ACC/AHA) guidelines [1]: mild (1.5 cm^2 ^< EOA ≤ 2.0 cm^2^), moderate (1.0 cm^2 ^< EOA ≤ 1.5 cm^2^) and severe (EOA ≤ 1.0 cm^2^).

#### Effective orifice area determination using transthoracic echocardiography

Transthoracic Doppler echocardiography (TTE) examinations were performed and analyzed by two experienced echocardiographers. TTE measurements were performed according to the American Society of Echocardiography guidelines [2] and included: LVOT diameter, LVOT flow velocity by pulsed-wave Doppler, transvalvular aortic jet velocity by continuous-wave Doppler and valve EOA using continuity equation [1]:

(1) $$\text{TT}{\text{E}}_{\text{CONT}}\text{EOA}=\text{S}{\text{V}}_{\text{LVOT}}/\text{VT}{\text{I}}_{\text{Ao}}=\left(\text{VT}{\text{I}}_{\text{LVOT}}\times {\text{A}}_{\text{LVOT}}\right)/\text{VT}{\text{I}}_{\text{Ao}}$$

Where SV_LVOT _is the stroke volume measured in the LVOT, A_LVOT _is the cross-sectional area of the LVOT calculated assuming a circular shape; and VTI_LVOT _and VTI_Ao _are the velocity-time integrals of the LVOT and transvalvular flow, respectively.

#### Cardiovascular magnetic resonance

**Figure 1:**
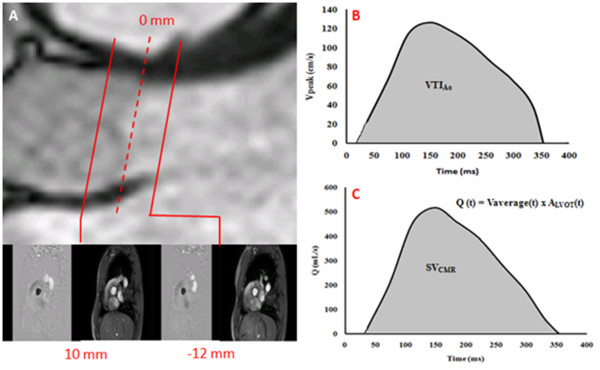
**Image planes used for CMR measurements**. The flow velocity map was acquired at two image planes: -12 mm upstream from aortic valve plane (used as the 0 mm reference) and +10 mm downstream of the aortic valve plane (Panel **A**). The cross-sectional area of the LVOT is measured at the -12 mm position. Panel B shows the instantaneous peak velocity (V_peak_) measurements in the cross-sectional aortic area at +10 mm. Panel C shows the instantaneous (Q) flow measurement at LVOT.

CMR studies were performed 2 to 4 weeks after TTE with patients in comparable hemodynamic state (Heart rate at TTE = 66 ± 11 bpm vs. CMR = 67 ± 12 bpm, p = NS). Imaging was performed with a clinical 1.5 Tesla Philips Achieva scanner operating release 2.6 level 3 and dedicated phased-array cardiac coil during successive end-expiratory breath-holds (Philips Healthcare, Best, The Netherlands). Imaging of cardiac function was performed by SSFP technique at 30 phases per cardiac cycle, with vectorcardiographic gating in 8-14 parallel short-axis, 2-chamber, 4-chamber, and 2 orthogonal LVOT planes (8 mm thickness, 0 mm gap). Typical parameters included TR/TE of 3.4/1.2 ms, flip angle 40°, NEX of 1, yielding in-plane spatial resolution of 1.6 × 2 mm. In addition, through-plane phase-contrast imaging was performed in the LVOT at 12 mm upstream from the aortic valve annulus (reference: 0 mm) and in the ascending aorta at +10 mm downstream of the annulus (Figure 1A). CMR imaging parameters consisted of: TR/TE of 4.60-4.92/2.76-3.05 ms, flip angle 15°, 24 phases, pixel spacing 1.32-2.07 mm, slice thickness 10 mm and acquisition matrix of 256 × 208. For each patient, peak aortic jet velocity measured by TTE was used to define CMR encoding velocity (CMR encoding velocity = (1.25 to 1.5) × peak jet velocity).

#### EOA determination using CMR

##### Effective orifice area using continuity equation

CMR images acquisitions and analyses were performed by investigators blinded to clinical and TTE results. The CMR-derived EOA (CMR_CONT_) was calculated using continuity equation [8,10,12]:

(2) $$\text{CM}{\text{R}}_{\text{CONT}}\text{EOA}=\text{S}{\text{V}}_{\text{CMR}}/\text{VT}{\text{I}}_{\text{Ao}}$$

Where SV_CMR _is the stroke volume derived from CMR velocities measured 12 mm upstream from the aortic valve (Simpson's rule was used to integrate flow during systole, Figure 1B) and VTI_Ao _(Figure 1C) is the velocity-time integral of the peak aortic flow velocity measured 10 mm downstream of the aortic valve during systole.

##### Effective orifice area by Shear Layer Detection

**Figure 2:**
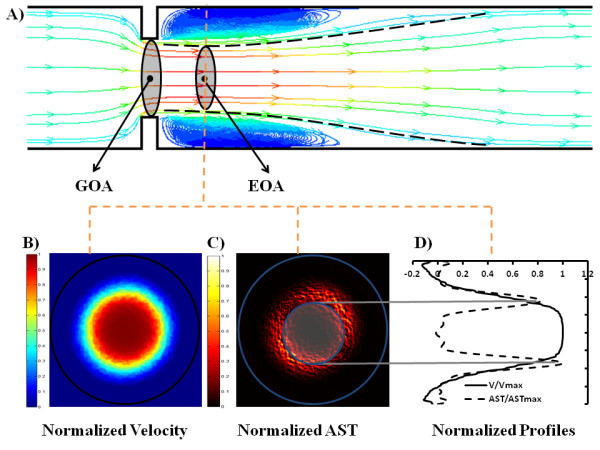
**Effective orifice area computed using shear layer detection method (JSLD)**. Effective orifice area computed using jet shear layer detection method (JSLD). Panel (**A**) shows flow streamlines through an orifice (geometric orifice area [GOA] = 1.66 cm^2 ^and effective orifice area [EOA] = 1.00 cm^2^). Dashed line represents a plane crossing the vena contracta (≈ 10 mm from the orifice). Panel (**B**) shows normalized velocity map at the vena contracta. Panel (**C**) shows AST shear layers computed from (**B**), JSLD identifies the inflexion points from the velocity profile corresponding to noise production, shear layers from vorticity and separation regions at the vena contracta position lead to EOA estimation (blue circle with transparency). Panel (**D**) shows the correspondence between normalized velocity profiles and normalized AST profiles, gray lines indicate the corresponding voxel on EOA identified shear layer

**Figure 3:**
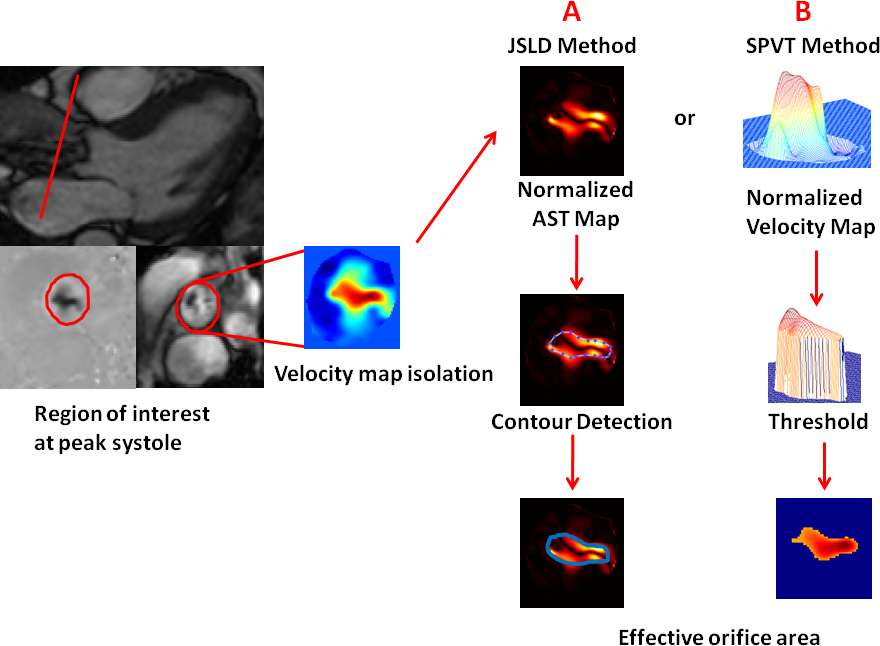
**Shear layer detection (JSLD) and single plane velocity truncation (SPVT) methods for the determination of EOA by CMR**. The velocity map is isolated along cardiac cycle by a semi-automatic detection of vessel borders. Once the velocity map at peak systole is isolated, both CMR_JSLD _and CMR_SPVT _methods can be applied. Panel A describes the CMR_JSLD _method: i) the isolated velocity map is used to compute normalized vorticity map and acoustical source term map, which is an amplification of vorticity map by local velocity magnitude; ii) a semi-automated algorithm that detects the peaks of AST shear layers corresponding to maximal noise production due to vorticity; iii) pixels inside the computed contour area are counted to estimate valve EOA. Panel B describes CMR_SPVT _method: i) the isolated velocity map is normalized with respect to its maximal velocity; ii) a threshold of 0.65 is used to truncate the normalized velocity profile; iii) pixels inside the threshold area are counted to estimate valve EOA. These algorithms could be also applied throughout systole.

This new method is based on the acoustical source term (AST) computed from the velocity field [14,29]. Briefly, flow disturbance and separation downstream of an aortic stenosis produce high vorticity field which is responsible for sound generation [24]. This concept of sound generated by flow is mainly based on the vortex sound theory developed first by Lighthill [30] and then by Powell [31] and Howe [32]. In this theory, the term [∇ (ωΛ*V*)], where ∇ is the nabla operator, ω is the vorticity field, and V is the velocity field, is called the acoustical source term (AST) and is responsible for the sound generated by unsteady fluid motion. This method provides an accurate and simple way of separating the jet-like zone from the recirculation zone right downstream of the stenotic valve and defines then the area of the vena contracta, i.e. the EOA [14] (Figure 2). This is due to the amplification of the vorticity by the magnitude of the local velocity. Only a single velocity profile at the peak systolic phase in the ascending aorta at 10 mm from aortic valve plane is necessary to determine the EOA with this method. This velocity profile was normalized with respect to peak velocity and then used to compute vorticity and AST shear layers profiles (Figure 2B and 2C). The CMR_JSLD _EOA was measured by a semi-automated algorithm that detects the peaks of normalized AST profiles corresponding to maximal noise production due to vorticity [14] (Figure 2D and 3A). An animation showing step-by-step how AST is determined from CMR velocity maps is included as Additional file 1.

##### Effective orifice area using single plane velocity truncation (SPVT) measurement method

**Figure 4:**
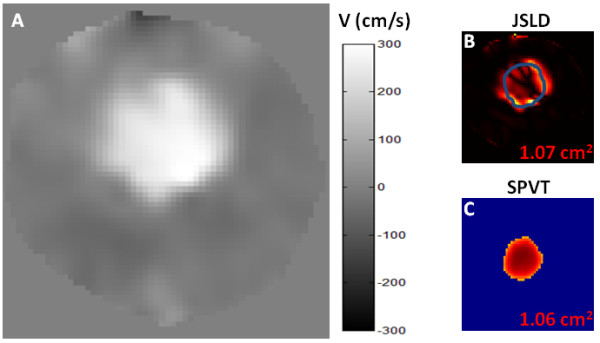
**Example of EOA determination using CMR_JSLD _and CMR_SPVT _methods in the in vitro study**. Panel A shows the isolated velocity map of a rigid plate orifice with GOA = 1.65 cm^2 ^and a theoretical EOA = 1.00 cm^2^. Panel B shows the EOA estimation using CMR_JSLD _method and panel C shows the EOA estimation using CMR_SPVT _method. B and C images are normalized from their maximal magnitude.

The same normalized velocity profile at peak systole used for CMR_JSLD _EOA computation is used for CMR_SPVT _EOA determination. However, instead of systematically computing vorticity and AST shear layer profiles (potentially resulting in truncation errors), the velocity profile is simply truncated at a threshold value of 0.65 and the area of the top surface obtained is considered to be the EOA. This threshold was obtained by a systematic analysis of AST shear layer profiles from the in vitro results (Figure 4). The same value was used in vivo for all patients (Figure 3B). A standalone application for computing the proposed EOA CMR methods can be found on our website http://users.encs.concordia.ca/~kadem/Research.html.

##### Measurement variability

To evaluate intra- and inter- observer variability related to image analysis by CMR and TTE, the measurements of EOA, using all methods, were repeated in a subset of 15 studies (11 AS patients and 4 control subjects) by two blinded observers with the use of the same set of TTE and CMR images. To further evaluate the intra- and inter- observer- variability related to image acquisition and analysis by TTE and CMR, 5 AS patients were imaged twice within 4 weeks (including image acquisition and analysis).

##### Statistical analyses

Results are expressed as mean ± SD. Paired 2-tailed Student's *t*-test was used to compare EOA measures. Correlations and agreement between CMR and TTE EOA measurements were assessed with the use of Pearson's correlation and Bland-Altman methods, respectively. Statistics were performed with SPSS 17 (SPSS, Chicago, IL).

## Results

### In vitro study

**Figure 5:**
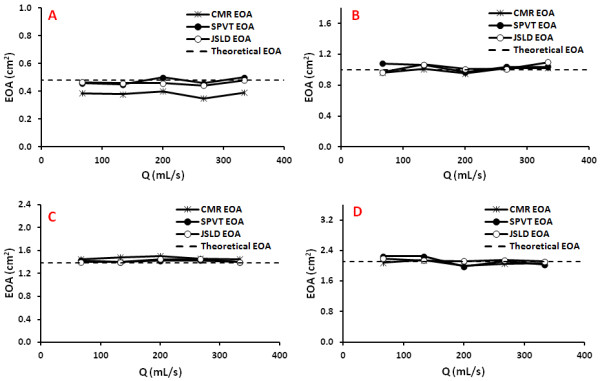
**Effective orifice areas obtained by the different CMR methods (continuity equation, JSLD, and SPVT) in the in vitro study under steady flow conditions**. Panels **A, B, C**, and **D **show the results obtained with an orifice of EOA of 0.48, 1.00, 1.38 and 2.11 cm^2^, respectively. The dashed line represents the EOA predicted by the potential flow theory (EOA = 0.61 × GOA); where GOA is the geometrical orifice area.

Figure 5 shows the results of EOA as a function of flow for the different orifices tested. EOA determined by CMR_CONT_, CMR_JSLD _and CMR_SPVT _methods were compared to the theoretical EOA predicted by the potential flow theory for sharp-edge orifices: EOA = 0.61 × GOA, where GOA is the geometric orifice area and the 0.61 is the contraction coefficient in the situation of sharp-edge orifices. Absolute and mean relative errors are reported in Table 1.

**Table 1:** Absolute and mean relative error for the determination of the EOA in the in vitro study

Effective Orifice area (cm^2^)	Method	Absolute error (cm^2^)	Mean relative error (%)
0.48	CMR_CONT_	0.10 ± 0.02	23 ± 5
	CMR_JSLD_	0.02 ± 0.01	4 ± 3
	CMR_SPVT_	0.02 ± 0.004	5 ± 1

1.00	CMR_CONT_	0.03 ± 0.01	3 ± 1
	CMR_JSLD_	0.04 ± 0.04	4 ± 4
	CMR_SPVT_	0.05 ± 0.02	5 ± 2

1.38	CMR_CONT_	0.09 ± 0.02	6 ± 2
	CMR_JSLD_	0.04 ± 0.03	3 ± 2
	CMR_SPVT_	0.04 ± 0.02	3 ± 1

2.11	CMR_CONT_	0.06 ± 0.03	3 ± 2
	CMR_JSLD_	0.03 ± 0.03	1 ± 1
	CMR_SPVT_	0.08 ± 0.07	4 ± 3

All orifices	CMR_CONT_	0.07 ± 0.04	9 ± 9
	CMR_JSLD_	0.03 ± 0.03	3 ± 3
	CMR_SPVT_	0.05 ± 0.04	4 ± 2

The reference value is the EOA predicted using potential flow theory (EOA = C_c _× GOA), where GOA is the geometrical orifice area and C_c _is the contraction coefficient that is = 0.61 (i.e. value for C_c _for harp-edge orifices with small aspect ratio). CMR_CONT_: EOA determined using continuity equation; CMR_JSLD_: EOA determined using shear layer detection (JSLD) based on acoustical source term concept; CMR_SPVT_: EOA determined using single plane velocity truncation (SPVT) method.

### In vivo study

Thirty-seven patients with mild to severe AS (71% men, age 61 ± 18 years) and eight healthy subjects (63% men, age 34 ± 8 years) underwent TTE and CMR studies. Valve morphology was bicuspid in twelve of the 37 (32%) AS patients and was indeterminate in 3 patients (8%) using TTE evaluation. Patient characteristics are given in Table 2.

**Table 2:** Patient Characteristics

Age (years)	61 ± 18
Male gender n (%)	32 (71)
Heart rate (bpm)	66 ± 11
Weight (kg)	76 ± 14
Height (cm)	169 ± 10
Body surface area (m^2^)	1.88 ± 0.21
Body mass index (kg/m^2^)	26 ± 4
Valve morphology	
Tricuspid n (%)	30 (67)
Bicuspid n (%)	12 (27)
Indeterminate n (%)	3 (6)

The table shows the mean ± SD or number of patients and percentage.

**Figure 6:**
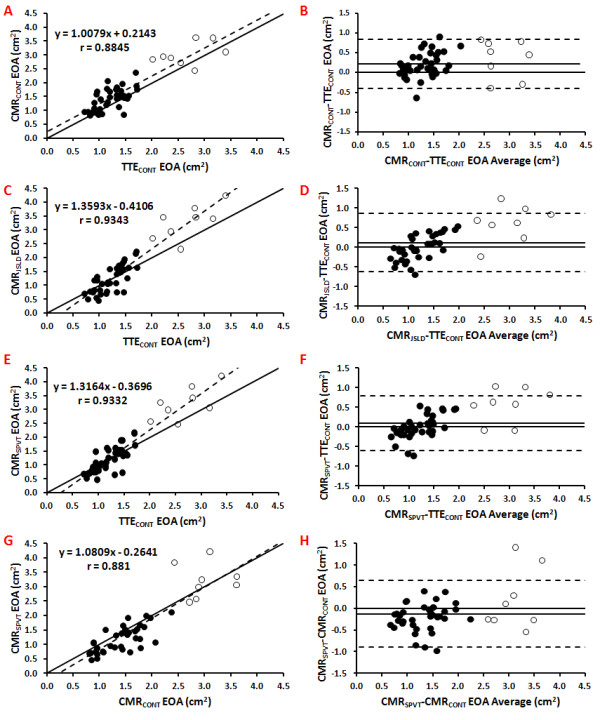
**Correlation and agreement between the EOAs obtained in vivo by the different CMR and TTE methods**. Panels A, C, E, and G show the Pearson correlation plots of CMR_CONT _vs. TTE_CONT_, CMR_JSLD _vs. TTE_CONT_, CMR_SPVT _vs. TTE_CONT_, and CMR_SPVT _vs. CMR_CONT_, respectively. The dashed line is the regression line and the solid line is the identity line. Panels B, D, F, and H show the Bland-Altman plots of CMR_CONT _vs. TTE_CONT_, CMR_JSLD _vs. TTE_CONT_, CMR_SPVT _vs. TTE_CONT_, and CMR_SPVT _vs. CMR_CONT_. The solid line is the mean bias and dashed lines are ± 1.96 standard-deviations lines. Non filled circles are healthy subjects.

Overall, there was a good correlation and concordance between the EOAs obtained by TTE_CONT _and those obtained by the 3 CMR methods. The average EOA was 1.46 ± 0.64 cm^2 ^for TTE_CONT_, 1.69 ± 0.73 cm^2 ^for CMR_CONT_, 1.57 ± 0.90 cm^2 ^for CMR_JSLD _and 1.58 ± 0.94 cm^2 ^for CMR_SPVT_. When compared to the EOA measured by TTE_CONT_, the results of correlation and agreement were r = 0.88, bias = +0.23 cm^2 ^and agreement limits:-0.39 and +0.84 cm^2 ^for CMR_CONT _(Figure 6A and 6B); r = 0.93, bias = +0.12 cm^2 ^and agreement limits:-0.62 and +0.86 cm^2 ^for CMR_JSLD _(Figure 6C and 6D); r = 0.93, bias = +0.10 cm^2 ^and agreement limits:-0.57 and +0.77 cm^2 ^for CMR_SPVT _(Figure 6E and 6F). There was also a good agreement between CMR_SPVT _and CMR_CONT _(r = 0.88, bias = -0.13 cm^2 ^and agreement limits:-0.90 and +0.65 cm^2^; Figure 6G and 6H).

In terms of clinical implications, seventeen (37%) patients had a change in AS severity class when using the EOA determined by CMR_CONT _instead of TTE_CONT_: three (6%) patients were re-classified in a more severe class and 14 (31%) in a less severe class. When using EOA determined by CMR_JSLD_: nineteen (42%) patients had a change in AS severity: six (13%) patients were re-classified in a more severe class and 13 (29%) in a less severe class. When using the EOA determined by CMR_SPVT_: twenty-one (46%) patients had a change in AS severity: eight (18%) patients were re-classified in a more severe class and 13 (29%) in a less severe class. Importantly, the severity was changed from severe to moderate in 2 patients with CMR_CONT _and in 3 patients with CMR_JSLD _or CMR_SPVT_.

### Measurement variability

The intra- and inter- observer variability of EOA measurements was 5 ± 5% and 9 ± 5% for TTE_CONT_, 2 ± 1% and 7 ± 5% for CMR_CONT_, 7 ± 5% and 8 ± 7% for CMR_JSLD_, 1 ± 2% and 3 ± 2% for CMR_SPVT_. When repeating image acquisition, reproducibility of measurements was 10 ± 8% and 12 ± 5% for TTE_CONT_, 9 ± 9% and 8 ± 8% for CMR_CONT_, 6 ± 5% and 7 ± 4% for CMR_JSLD _and 3 ± 2% and 2 ± 2% for CMR_SPVT_, for observer one and two respectively.

## Discussion

Contemporary clinical evaluation of the AS severity is mainly based on the TTE measurements of valve EOA, which corresponds to the minimal cross-sectional area of the transvalvular flow jet downstream of the aortic valve. However, TTE measurements are sometimes not feasible or might lead to discordant results. In particular, the situation where the EOA measured by TTE is in the severe range (e.g. 0.8 cm^2^) but the gradient (or other stenotic indices) is in the moderate range (i.e. 30 mmHg) poses a challenge for the treating physician, especially if the patient is symptomatic. This discordance may be due to measurement errors, small body size, or low flow state conditions [33,34]. Low flow state conditions may occur in the setting of a low LV ejection fraction (LVEF) but also in the context of preserved LVEF. This later condition, named paradoxical low flow AS [35] occurs in patients with pronounced concentric LV remodelling, small LV cavities and impaired LV filling and is characterized by reduced pump function and thus reduced stroke volume and transvalvular flow rate despite preserved LVEF. In patients with low flow states, the transvalvular gradient, which is highly flow-dependent, may be pseudo-normalized and may thus underestimates the severity of AS.

In these situations where the TTE measurements are not feasible or discordant, it is necessary to use another imaging modality to determine the actual AS severity and to confirm or infirm the results of TTE. This information is crucial for therapeutic decision making.

In the present study, we proposed a new method based on direct determination of the valve EOA from a single velocity measurement downstream of the stenosis using AST jet shear layer detection. The results of this study reveal an excellent agreement between the EOA estimated by this new method and the EOA predicted in vitro by the potential flow theory or measured in vivo by TTE with the continuity equation method. We also proposed a simplified version of the JSLD method, which does not require the computation of the vorticity term (included in the definition of the AST).

The main advantage of these methods is that they are simple and require only one image plane and one measurement to calculate the EOA. This minimal requirement for the determination of the EOA contributes to the reduction of the errors and may, at least in part, explain why they have better inter- and intra- observer variability compared to the other TTE or CMR methods. It is also important to note that Yap et al. [12] have previously introduced a method requiring a single measurement plane by measuring the stroke volume at the level of the aorta, instead of the LVOT. The main originality and interest of the new methods we are proposing in our paper is that the determination of the EOA only requires a single plane velocity measurement and moreover it does not require measurement of stroke volume.

**Figure 7:**
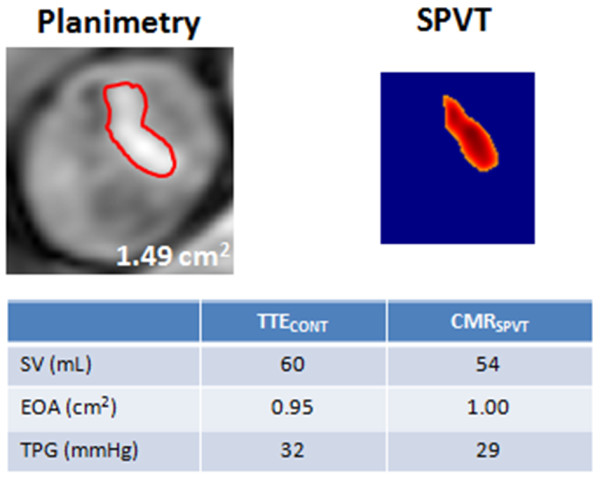
**Example of a patient with discordant echocardiography findings in whom stenosis severity was corroborated by the measurement of EOA with the use of CMR_SPVT_**. EOA: effective orifice area; SV: stroke volume; TPG: transprosthetic gradient.

The new simple and reliable methods described in the present study may thus help to confirm stenosis severity and guide therapeutic management. In this regard, Figure 7 shows the case of a symptomatic patient who had a discordance between EOA (0.95 cm^2^) and mean gradient (32 mmHg) at TTE, thus raising uncertainty about the actual severity of the stenosis. CMR_SPVT _confirmed that the EOA is in the severe range (1.00 cm^2^) and the patient was referred to surgery, which revealed a heavily thickened and calcified valve.

**Figure 8:**
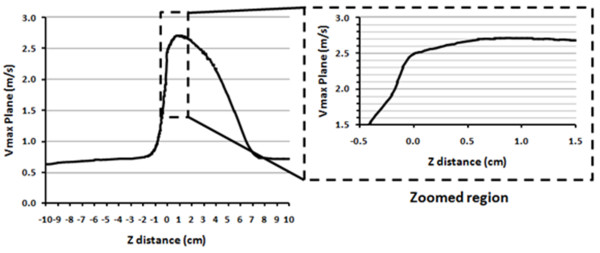
**Vena contracta position estimated from numerical simulations**. Three-dimensional numerical simulations of a steady flow (20 l/min) through an orifice plate with an EOA = 1.00 cm^2 ^were performed using a computational fluid dynamics package (Fluent, ANSYS, Canonsburg, PA, USA) package. More than 10^6 ^elements were used. Turbulent flow was modelled using a standard k-ω model. The figure shows the maximum velocity along the centre of orifice from -10 cm to +10 cm. Zoomed region shows the region of vena contracta downstream of the sharp-edge orifice.

**Figure 9:**
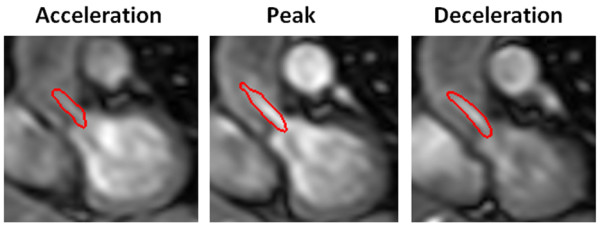
**Vena contracta region during systole on a severe aortic stenosis (EOA = 0.88 cm^2^)**. Vena contracta region (in red) remained almost constant closer to the aortic valve. Vena contracta exact position was 13 mm from the aortic valve at peak systole. Vena contracta region was defined using JSLD method.

Sondergaard et al. [7,36] have introduced a similar approach as the one proposed in the present study to determine the GOA (not the EOA) of a stenotic valve using the velocity map. This was achieved by performing the measurements at the level of the aortic valve and by using a threshold of 0.5 × maximal velocity. Their method was validated in vitro and they found a very good concordance with the actual geometric area of the rigid circular orifices used in their study. In the present study, the purpose of the new proposed methods is to estimate the EOA. Hence, an optimal location of the CMR single plane measurement is of primary importance in order to minimize volume averaging errors. For all the cases investigated in this study, the measurements were performed 10 mm downstream of the aortic valve annulus. This choice was motivated by the results obtained through numerical simulations showing that for a rigid circular orifice of effective orifice area of 1.00 cm^2 ^(cut-off value of EOA for severe AS), performing the measurements with a slice thickness of 10 mm (averaging the velocity profiles within the slice thickness) at 10 mm downstream of the valve annulus does not yield to significant differences compared to measurements performed exactly at the location of the vena contracta position (Figures 8 and 9). This distance is in agreement with other in vitro tests performed under pulsatile flow conditions [37]. It is important however to note that inadequate positing of the slice, typically farther from the aortic valve plane, will lead to an overestimation of the EOA by all CMR methods: EOA based on the continuity equation, JSLD method and SPVT method. The resulting overestimation, compared to the TTE derived EOA, is proportional to the ratio of aortic cross-sectional area/EOA, or to valve energy loss coefficient.

### Study limitations

The main limitations of this study are: i) small number of patients with severe AS; ii) absence of gold-standard reference method for EOA measurement in vivo. The determination of valve EOA can be performed by catheterization using the Gorlin formula. However, this method is invasive and not without risk for the patients [38]. Furthermore, it has important limitations and thus cannot be considered as a gold standard reference method [39].

Another limitation of this study is the potential effect of aliasing on the EOA determined by all the methods. Aliasing may affect flow measurements and EOA proposed methods leading to a systematic overestimation of EOA. Interestingly, EOA obtained using JSLD method will not be affected by aliasing as long as the velocity profile is not truncated below its inflexion points. This represents an extreme case in clinical practice. Finally, it should be mentioned that some unwrapping algorithms [40-43] allow the correction of aliasing and it is generally avoided in clinical practice.

## Conclusion

There was an excellent agreement between the EOA estimated by the CMR_SPVT _method and: 1) the theoretical EOA in vitro, and 2) the TTE_CONT _EOA in vivo. Furthermore, the CMR_SPVT _method was superior to the other TTE or CMR methods in terms of measurement variability. This new simple and non-invasive method may be helpful to corroborate stenosis severity in patients for whom Doppler-echocardiography exam is inconclusive.

## Competing interests

The authors declare that they have no competing interests.

## Authors' contributions

All authors contributed to the scope and outline of the manuscript. JG wrote the final draft. All authors read and approved the final manuscript.
